# Dietary capsaicin normalizes CGRP peptidergic DRG neurons in experimental diabetic peripheral neuropathy

**DOI:** 10.1038/s41598-021-81427-w

**Published:** 2021-01-18

**Authors:** Xiao-Yi Zhang, Zheng Guo, Tu-Ping Li, Tao Sun

**Affiliations:** 1 Department of Anesthesiology, Shanxi Medical University, 86 Xinjiannan Road, Taiyuan, 030001 Shanxi China; 2 Department of Anesthesiology, Second Hospital of Shanxi Medical University, 382 Wuyi Road, Taiyuan, 030001 Shanxi China; 3 Key Laboratory of Cellular Physiology (Shanxi Medical University), National Education Commission, Shanxi Medical University, 86 Xinjiannan Road, Taiyuan, 030001 Shanxi China

**Keywords:** Diseases of the nervous system, Neurodegeneration, Diseases, Diabetes complications

## Abstract

Diabetic sensory neuropathy leads to impairment of peripheral sensory nerves and downregulation of calcitonin gene-related peptide (CGRP) in a functionally specific subset of peripheral sensory neurons mediating pain. Whether CGRP plays a neuroprotective role in peripheral sensory nerve is unclear. We evaluated alterations in noxious thermal sensation and downregulation of CGRP in the 8 weeks after induction of diabetes in rats. We supplemented capsaicin in the diet of the animals to upregulate CGRP and reversed the downregulation of the neuropeptide in the dorsal root ganglion (DRG) neurons dissociated from the diabetic animals, via gene transfection and exogenous CGRP, to test disease-preventing and disease-limiting effects of CGRP. Significant preservation of the nociceptive sensation, CGRP in spinal cord and DRG neurons, and number of CGRP-expressing neurons was found in the diabetic animals given capsaicin. Improvement in the survival of the neurons and the outgrowth of neurites was achieved in the neurons transfected by LV-CGRP or by exogenous CGRP, paralleling the correction of abnormalities of intracellular reactive oxygen species and mitochondrial transmembrane potentials. The results suggest that downregulation of CGRP impairs viability, regeneration and function of peripheral sensory neurons while capsaicin normalizes the CGRP peptidergic DRG neurons and function of the sensory nerves.

## Background

Diabetic peripheral neuropathy (DPN) characterized by abnormality and even loss of sensation of pain, resulting from impaired neuronal soma and axonal regeneration^1,2^, affects up to 50% of diabetes patients^3^. Current therapeutic treatment options for DPN are very limited. Importantly, DPN can eventually lead to some lethal pathological consequences, such as silent myocardial ischemia^4^. But the mechanism fundamentally critical to the development of DPN is not completely understood. Lines of evidence have implicated the insufficiency or tolerance to insulin, hyperglycemia, hypoxia and inflammation^5,6^ may be associated with the formation and progress of the peripheral neuropathy. The pathology may be associated with increases in production of reactive oxygen species (ROS), oxidative stress and mitochondrial dysfunction in peripheral sensory neurons^5^ and the Schwann cells in the axons^7^, which further lead to degeneration of the peripheral nerve fibers, impairment of the dorsal root ganglion (DRG) neurons and hypoalgesia.

Among the primary sensory neurons, the small and medium diameter neurons are responsible for transmission of pain or nociceptive signals. The neuropeptides, calcitonin gene-related peptide (CGRP) and substance P (SP), are important co-transmitters mediating the pain-related signals from a subset of Aδ and C axons^8,9^. Transient receptor potential vanilloid 1 (TRPV1), a non-specific cation channel whose activation leads to release the neuropeptides^10^. Significantly lower levels of CGRP and SP in the serum of diabetic patients were reported by Wang and colleagues^11^ and our group^12^. We have previously found a close correlation of development of the impairment of noxious thermal sensation with the significant reductions of CGRP and SP in the DRG neurons in the experimental animals^13,14^, while dietary supplementary capsaicin, a natural agonist of TRPV1 significantly preserved CGRP in the DRG neurons of diabetic animals^15^. Although effects of CGRP in modulation of cytosolic and mitochondrial calcium, ROS, mitochondrial transmembrane potentials and myelin sheath were reported^16–18^, it is still unknown whether CGRP plays a neuroprotective role in the development of diabetic peripheral neuropathy.

We decided to use a diabetic rat model of peripheral neuropathy based on this clinical scenario and analyzed the correlation of the desensitization of the nociceptive sensation with the reduction of CGRP and the CGRP-expressing neurons in the DRG of the animals and then evaluated the disease-limiting effect of CGRP, by pharmacological and genetic modulating approaches. This study was conducted based on the hypotheses. The first hypothesis is that under normal conditions, CGRP participates in nociceptive signal transduction and maintenance of the viability of the peripheral sensory neurons via reduction of ROS and maintenance of mitochondrial transmembrane potentials (MMP). Downregulation of CGRP causes imbalance of mitochondrial metabolism of ROS and the transmembrane potentials and leads to impairment of the sensory neurons and the nociceptive sensation. Our second hypothesis is that restoration of CGRP, via chronic activation of TRPV1 by dietary capsaicin, genetic modulatory and pharmacological approaches, would limit the abnormality of intracellular ROS and the mitochondrial transmembrane potentials, and improve the impairment of the peripheral nerves in the development of diabetic neuropathy.

## Results

### Characteristics of the diabetes model

**Figure 1:**
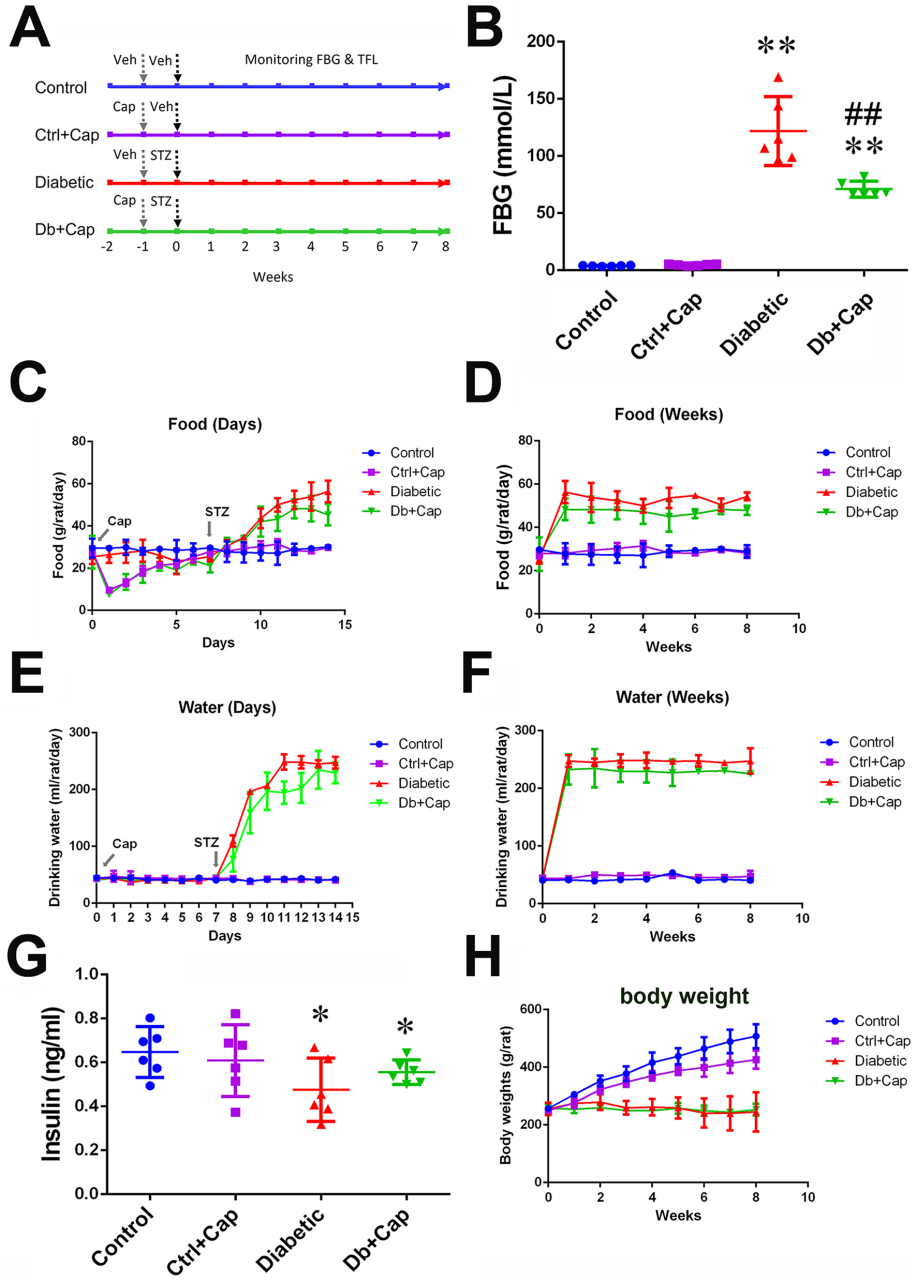
Diabetic model. (**A**) Profile of in vivo experiments in the four groups of animals, two diabetic groups, one treated with capsaicin (Db + Cap) and another without the treatment (Db), and two non-diabetic control group, one without and another with capsaicin treatment (Ctrl and Ctrl + Cap). Timing of treatments with capsaicin (Cap), streptozotocin (STZ) and the solvent of capsaicin (veh) and STZ (Veh) is indicated by the arrows. (**B**) Chronic activation of TRPV1 by dietary capsaicin significantly inhibits the elevation of fasting blood glucose (FBG) at the end of the 8 weeks after the treatment with STZ. (**C**) Significant increases in food consumption in the first 7 days and in the following 7 weeks (**D**) were observed in the diabetic animals including the capsaicin treated diabetic animals (Cap indicates the start timing of the capsaicin treatment and STZ, the timing of injection of STZ). (**E**) The significant increases in drinking water in the first 7 days after the treatment of STZ (labeled as STZ) and in the following 7 weeks (**F**) in the diabetic animals including the capsaicin treated (Cap indicates the start timing of capsaicin treatment). (**G**) The serum insulin was significantly lower in the diabetic and capsaicin treated diabetic animals, at the end of the 8 weeks after injection of STZ. (**H**) Significantly lower body weights were detected in the diabetic animals, with and without the capsaicin treatment and in the non-diabetic animals treated with capsaicin. *Control or Ctrl* non-diabetic control group, *Ctrl + Cap* non-diabetic animals treated with capsaicin, *Diabetic* diabetic group, *Db + Cap* diabetic animals treated with capsaicin. *p < 0.05, compared to the control; **p < 0.01, compared to the control; ^##^*p* < 0.01, compared to diabetic.

The data reported here were collected from the animals presenting significantly high levels of blood glucose (> 16.7 mmol/L) 24 h after injection of STZ and throughout the 8 weeks of the development of diabetes. Significant increases in the consumption of food and drinking water (Fig. 1B–F) with lower levels of serum insulin and body weights (Fig. 1G,H) were observed in the diabetic animals, when compared with the age-matched controls. A significant smaller increase in fasting blood glucose was observed in the diabetic animals treated with dietary capsaicin (Fig. 1B) with non-significant restoration of normal serum insulin levels, when compared with the diabetic animals without the treatment of capsaicin (Fig. 1G). It was observed in this study that the animals first avoided the food but then became acquainted, which might indicate the concentration of capsaicin in the food was high enough being tasted stimulating (hot and spicy) to the animals. In the meantime, rapid desensitization of the local trigeminal nerve endings of the oral mucosa might occurred under direct stimulation by the capsaicin in the food, facilitating the later increase in food consumption. Moreover, a significantly smaller gain in body weight was observed in the non-diabetic animals treated with capsaicin (Fig. 1H) than in the non-diabetic animals not treated with capsaicin.

### Dietary capsaicin improved hypoalgesia in developing diabetes

**Figure 2:**
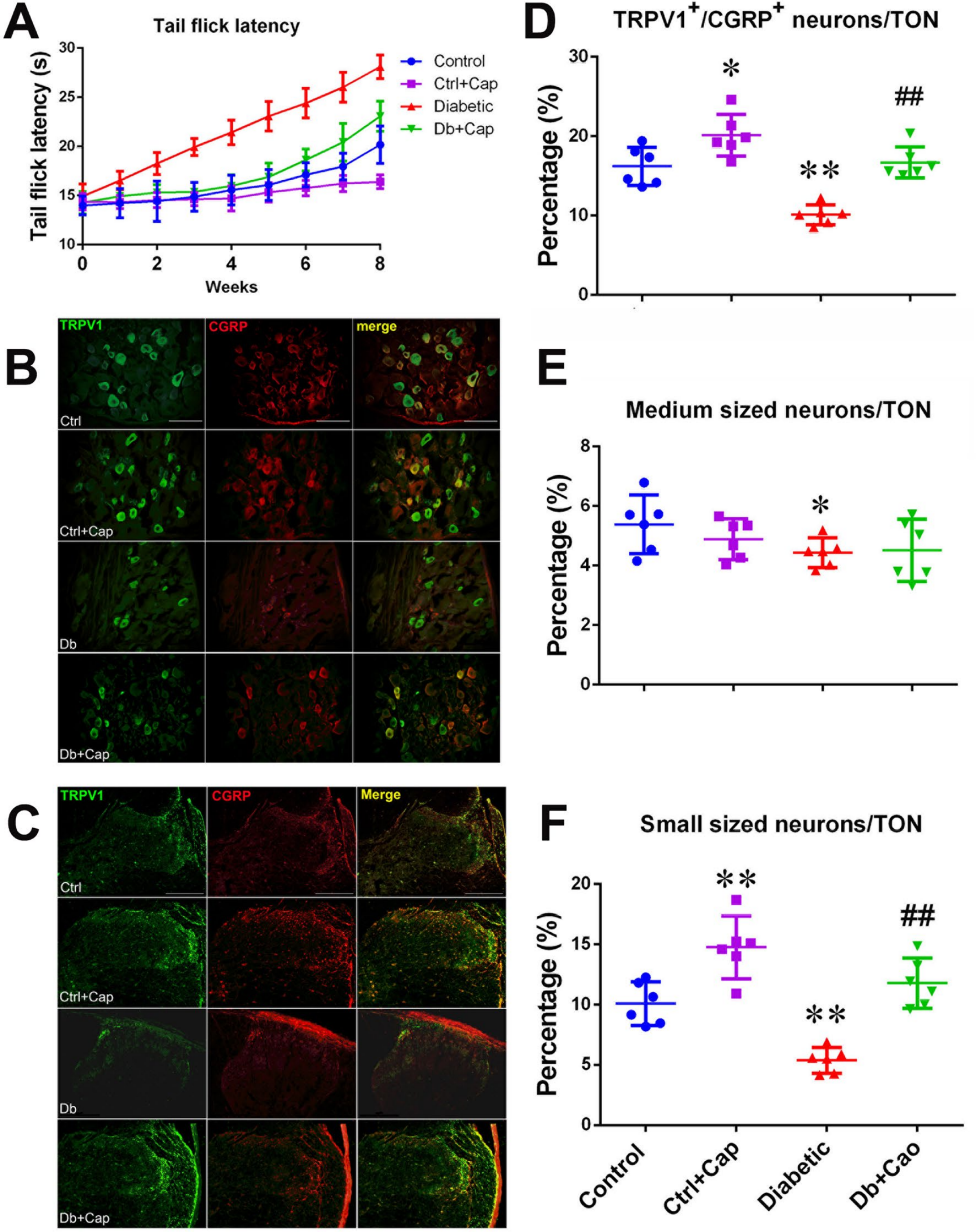
Preservation and impairment of the peripheral sensory nerves. Significant attenuation of the elevation of tail flick latency (**A**) was observed paralleling the preservation of CGRP-expressing DRGs (**B**) and in the spinal dorsal horn (**C**), at the 5th lumber segment of the animals from the capsaicin treated diabetic group, stained with antibodies for TRPV1 (in green) and CGRP (in red) and the mergers (in yellow), showing massive losses of the expressions of TRPV1 and CGRP in the spinal dorsal horn, especially in the superficial laminae (marked by the circle) at the end of the 8 weeks after induction of diabetes. The preservation of the DRG neurons co-expressing TRPV1 and CGRP (**D**), the medium diameter neurons (**E**) and the small diameter neurons (**F**). *Control or Ctrl* non-diabetic control group, *Ctrl + Cap* non-diabetic animals treated with capsaicin, *Diabetic or Db* diabetic group, *Db + Cap* diabetic animals treated with capsaicin, *TON* total observed neurons. *p < 0.05, compared to the control; **p < 0.01, compared to the control; ^##^*p* < 0.01, compared to the diabetic. The scales = 100 µm.

There was no difference in values of tail flick latency among the animals in the groups of control, control treated with capsaicin (Ctrl + Cap), diabetic and diabetic treated with capsaicin (Db + Cap, all *p* > 0.05), when tested at the time of STZ treatment. In the 8 weeks following the treatment with STZ, significant elevations of tail flick latency, from the values of 14.9 ± 1.2 s (tested at the time of STZ treatment) up to 28.1 ± 1.2 s (by 89.9% ± 23.4%), were detected in the diabetic animals (n = 6), while a significantly less increases in the latency, from 14.2 ± 1.1 s up to 23.0 ± 1.5 s (by 62.3% ± 15.0%), were obtained in the diabetic animals treated with capsaicin supplement (n = 6, all *p* < 0.01, Fig. 2A). The same effect was found in the non-diabetic animals treated with capsaicin (Ctrl + Cap), when compared to the TFL in the controls not treated with capsaicin (Fig. 2A). The difference in elevation of the tail flick latency between the animals with and without capsaicin supplement indicates that chronic treatment of capsaicin may sensitize the individual neurons, recruit the new neurons and/or be neuroprotective in the animals chronically exposed to capsaicin (Fig. 2A).

### Dietary capsaicin preserved TRPV1 and CGRP co-expressing small diameter DRG neurons

Six dorsal root ganglia were dissected from the 5th lumbar or the first sacral segment of six animals from the experimental groups including diabetic animals (Diabetic, n = 6), the capsaicin treated diabetic animals (Db + Cap, n = 6), the non-diabetic animals (Control, n = 6) and capsaicin treated non-diabetic animals (Ctrl + Cap, n = 6). The results showed significant reductions of the number of small and medium diameter neurons co-expressing TRPV1/CGRP in the DRG of the diabetic animals (control vs diabetic: in small neurons, 10.1 ± 1.7% vs 5.4 ± 1.0%, *p* < 0.01; in medium neurons, 5.4 ± 0.9% vs 4.4 ± 0.5%, *p* < 0.05; Fig. 2B,D–F). Reductions in immunoreactivity for TRPV1 and CGRP in the shallow laminae of the spinal dorsal horn were also observed (Fig. 2C). However, definite preservation of the TRPV1/CGRP co-expressing small diameter neurons in the DRGs was seen in the capsaicin treated diabetic animals (Diabetic vs Db + Cap, 5.4 ± 1.0% vs 11.8 ± 2.0%, *p* < 0.01, Fig. 2D–F). A similar effect was detected in the non-diabetic animals treated with capsaicin, presenting a significant increase in the proportion of small diameter neurons immunoreactive for TRPV1 and CGRP (Control vs Ctrl + Cap, 10.1 ± 1.7% vs 14.7 ± 2.5, *p* < 0.01). The observations indicate that capsaicin promotes expression of TRPV1 and CGRP in DRG neurons, especially in the small diameter neurons and in the spinal dorsal horn (Fig. 2B–F). The analysis of the digitized fluorescence immunoreactive for TRPV1 and CGRP showed the same trends of the significant changes of TRPV1 and CGRP in the DRGs and spinal cord (Table S1, in supplementary data). But the proportions of small sized neurons immunoreactive for TRPV1 and CGRP were increased in the same magnitude as the magnitude of reduction of the small neurons not immunoreactive for TRPV1 and CGRP (Tables S2 and S3, in supplementary data). The results indicate that the capsaicin treatment normalized the proportions of the TRPV1/CGRP-expressing small diameter neurons in the DRG.

### Dietary capsaicin preserved CGRP and TRPV1 in the DRG and the spinal dorsal horn

**Figure 3:**
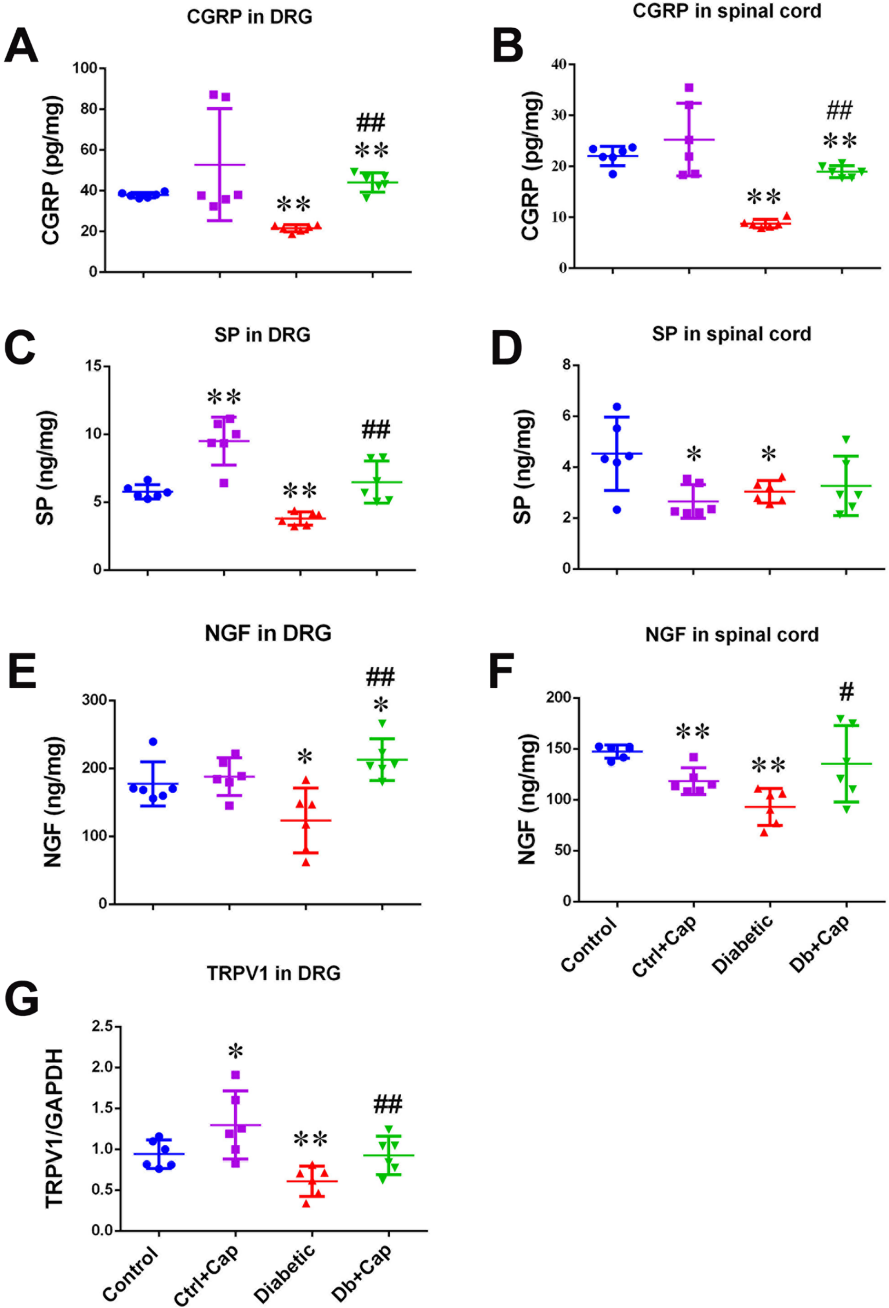
Preservation of CGRP, SP, NGF and TRPV1 in the DRGs and the spinal cord. Significant preservation of CGRP, SP, NGF and TRPV1 in the DRGs (**A**,**C**,**E**,**G**, respectively) and CGRP and NGF in the spinal cord (**B**,**D**,**F**, respectively), at the 5th lumber segment, was detected in the capsaicin treated diabetic animals. In the spinal cord, only CGRP was preserved in the non-diabetic animals treated with capsaicin. *Control* non-diabetic control group, *Ctrl + Cap* non-diabetic animals treated with capsaicin, *Diabetic* diabetic group, *Db + Cap* diabetic animals treated with capsaicin. *p < 0.05, compared to the control; **p < 0.01, compared to the control; ^#^*p* < 0.05, compared to diabetic; ^##^*p* < 0.01, compared to diabetic.

The ELISA and the Western blot assay revealed significant reductions in CGRP, SP, NGF and TRPV1 in the DRG and the spinal cord in the 5th lumbar or the first sacral segment were observed in the diabetic animals (n = 6, Fig. 3A–G). The observation, along with the findings of reductions of the number of TRPV1/CGRP expressing small neurons, the expressions of immunoreactive materials for the molecules in the dorsal horn and elevation of tail flick latency, strongly suggests a close association of the abnormality of the molecules with impairment of the primary sensory neurons and sensory functions in diabetic animals. Chronic treatment of the diabetic animals with capsaicin, significantly preserved CGRP, SP, NGF and TRPV1 in the DRG neurons (Fig. 3A,C,E,G) and CGRP, NGF and TRPV1 in the spinal cord (Fig. 3B,D,F, Table S). The findings present a close association of the normalization of expressions of the TRPV1 and CGRP in the DRG and spinal cord (Fig. 3A,G, Table S) with the preservation of the TRPV1/CGRP-expressing small neurons and the sensory sensitivity to the noxious heating stimulation in the capsaicin treated diabetic and non-diabetic animals (Fig. 2A–F), indicating that transduction occurred.

### Transfection of LV-CGRP upregulated CGRP and TRPV1 in DRG neurons

#### Effects on CGRP and the coding mRNA

**Figure 4:**
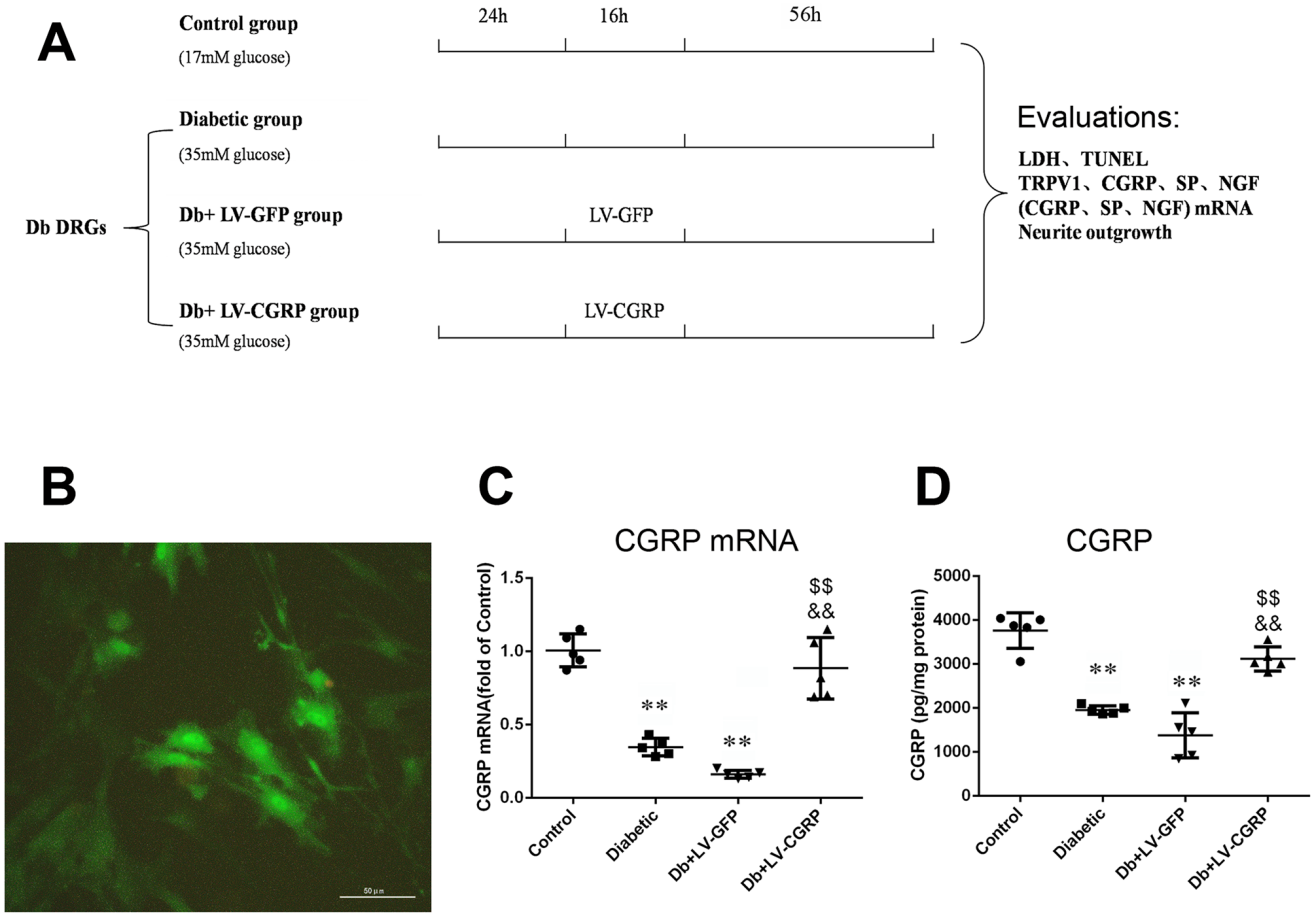
Transfection of CGRP genes restored CGRP and the coding mRNA. (**A**) Profile of the in vitro experiments in dissociated cultured DRG neurons collected from the diabetic animals, at the end of the 8th week after induction of diabetes. The concentrations of glucose are labeled in the bracket under each group. Success of transfection of the vectors was primarily evaluated by visualization of green fluorescence in the neurons (**B**). Transfection of CGRP genes completely reversed the downregulations of CGRP coding mRNA (**C**) and CGRP (**D**). *Control* the neurons from the age matched non-diabetic animals, *Diabetic* the neurons from the diabetic animals, *Db + LV-GFP* DRG neurons from diabetic animals transfected with GFP genes, *Db + LV-CGRP* DRG neurons from diabetic animals transfected with CGRP genes. **p < 0.01, compared to the control; ^$$^*p* < 0.01, compared to Diabetic group; ^&&^p < 0.01, compared to Db + LV-GFP group. The scale = 50 µm in (B).

We transfected the DRG neurons dissociated from the diabetic animals after 8 weeks of the injection of STZ with LV-CGRP (Fig. 4B), to test an independent role of CGRP in the neuroprotection. The transfection of LV-CGRP completely reversed the downregulation of the coding mRNA and the proteins of CGRP in the DRG neurons (Fig. 4C,D). Conversely, no significant change in CGRP or its coding mRNA was detected in the neurons transfected by LV-GFP. The results demonstrated a possibility and a viability of restoration of the levels of the CGRP in the DRG neurons of the animals after 8 week-long development of diabetes.

#### Changes in TRPV1 and the coding mRNA

**Figure 5:**
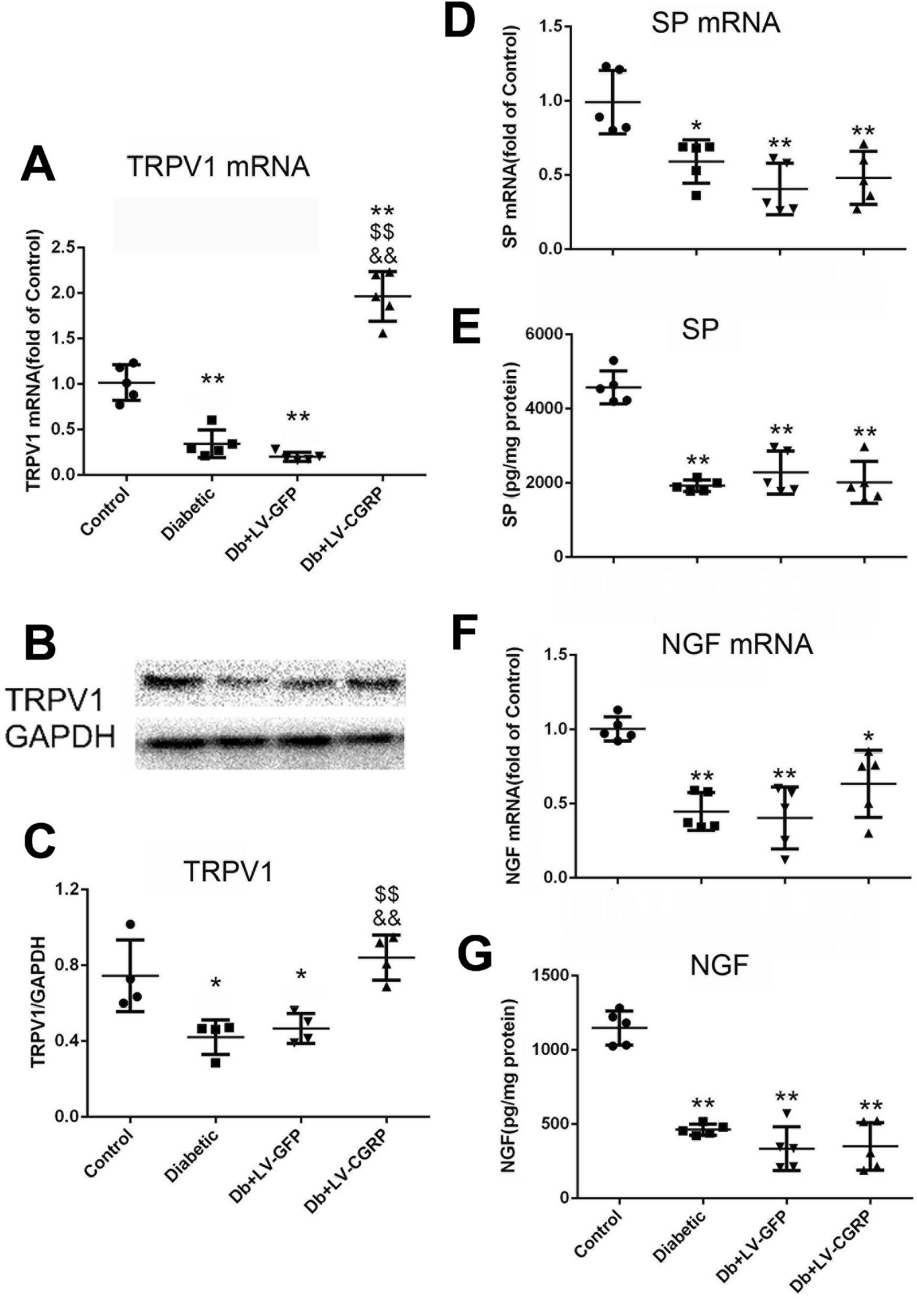
Transfection of LV-CGRP restored TRPV1 without alteration of SP and NGF. Transfection of LV-CGRP in the DRG neurons from diabetic animals completely restored TRPV1 coding mRNA (**A**) and TRPV1 (**B**,**C**) while no alteration was detected in SP (**D**,**E**) and NGF (**F**,**G**). *Control* the neurons from the age matched non-diabetic animals, *Diabetic* the neurons from the diabetic animals, *Db + LV-GFP* DRG neurons from diabetic animals transfected with GFP genes, *Db + LV-CGRP* DRG neurons from diabetic animals transfected with CGRP genes. *p < 0.05, compared to the control; **p < 0.01, compared to the control; ^$$^*p* < 0.01, compared to diabetic group; ^&&^*p* < 0.01, compared to Db + LV-GFP group.

Surprisingly, transfection of CGRP genes in the neurons from the diabetic animals also fully restored the levels of TRPV1 and its coding mRNA (Fig. 5A–C), with no change in SP and NGF (Fig. 5D–G).

### Transfection of LV-CGRP and exogenous CGRP inhibited lactic dehydrogenase (LDH) and neuron apoptosis

**Figure 6:**
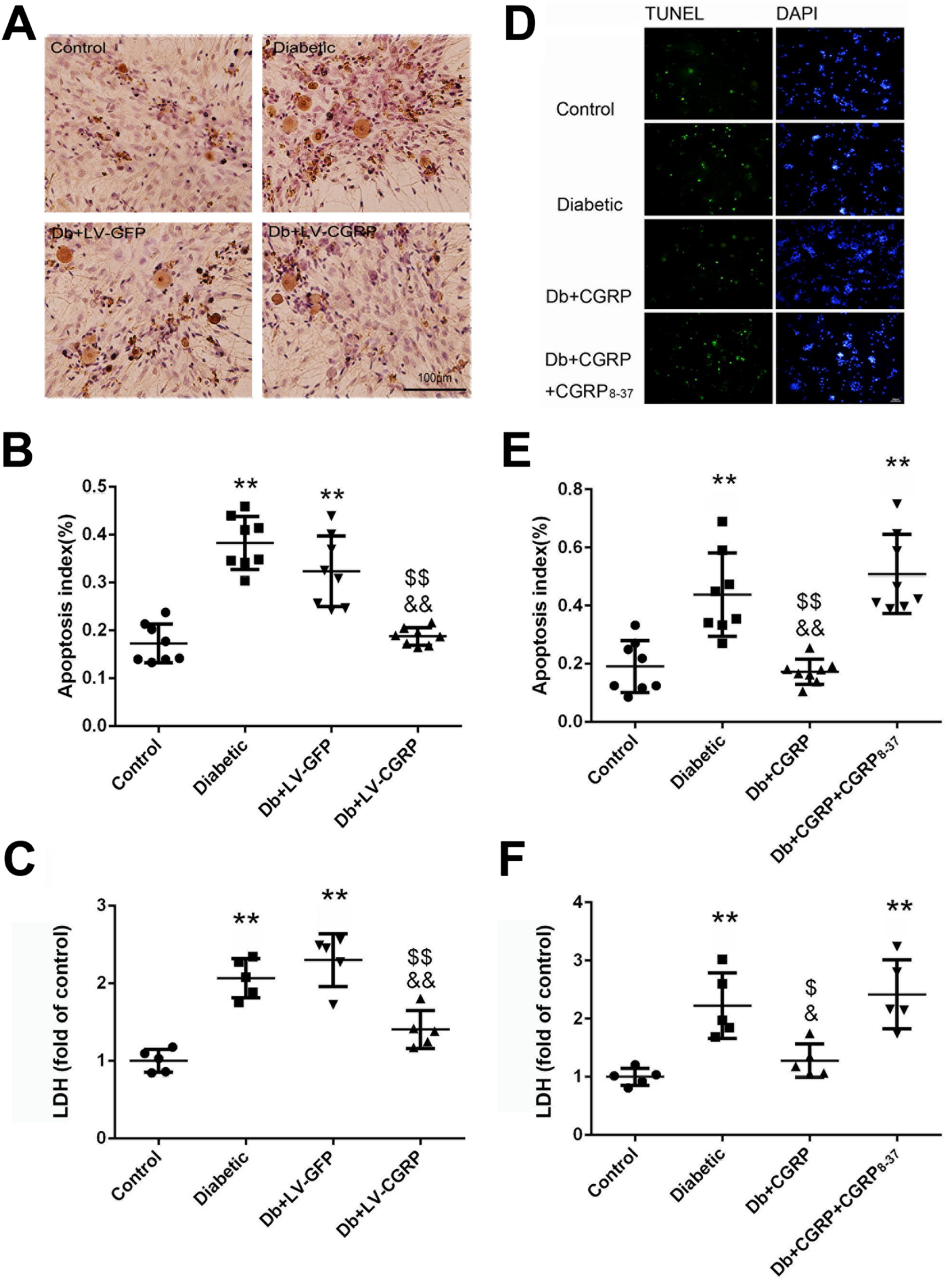
Transfection of LV-CGRP and exogenous CGRP alleviated neuronal injury of the DRG neurons. Transfection of CGRP genes effectively alleviated the apoptotic changes of the dissociated DRG neurons from the diabetic animals (**A**,**B**) and reduced the elevation of LDH in the culture medium (**C**). Exogenously administered CGRP (at 10^−8^ mol/L, the final concentration) produced the same protective effects on the apoptotic changes (**D**,**E**) and the LDH (**F**). The effects of CGRP were completely reversed by CGRP_8–37,_ a specific antagonist of CGRP receptor (at 10^−7^ mol/L, the final concentration). *Control* the neurons from the age matched non-diabetic animals, *Diabetic* the neurons from the diabetic animals, *Db + LV-GFP* DRG neurons from diabetic animals transfected with GFP genes, *Db + LV-CGRP* DRG neurons from diabetic animals transfected with CGRP genes. *Db + CGRP* the neurons collected from the diabetic animals treated with CGRP, *Db + CGRP + CGRP*_*8–37*_ the neurons collected from the diabetic animals treated by CGRP_8–37_ then by CGRP. **p < 0.01, compared to the control; ^$^*p* < 0.05, compared to diabetic group; ^$$^*p* < 0.01, compared to Diabetic group; ^&^*p* < 0.05, compared to Db + CGRP + CGRP_8–37_ group; ^&&^*p* < 0.01, compared to Db + LV-GFP group or Db + CGRP + CGRP_8–37_ group. The scales = 100 µm in (**A**) and 50 µm in (**D**), respectively.

A marked anti-apoptotic effect was achieved in the cultured neurons dissociated from the diabetic animals (Fig. 6A,B), by transfection of CGRP genes. The rise of LDH in the culture medium of the neurons from the diabetic animals was significantly inhibited by the gene transfection (Fig. 6C). Whereas transfection of LV-GFP did not show any effect on the viability of the neurons and LDH in the culture.

Similarly, exogenously administered CGRP (at a final concentration of 10^–8^ mol/L) significantly attenuated the neuron apoptotic changes and the elevation of LDH in the culture medium of the neurons (Fig. 6D–F). The effects were completely abolished by CGRP_8–37_, indicating the specific receptor mediates protective effect of CGRP.

### Transfection of LV-CGRP and exogenous CGRP improved neurite outgrowth

**Figure 7:**
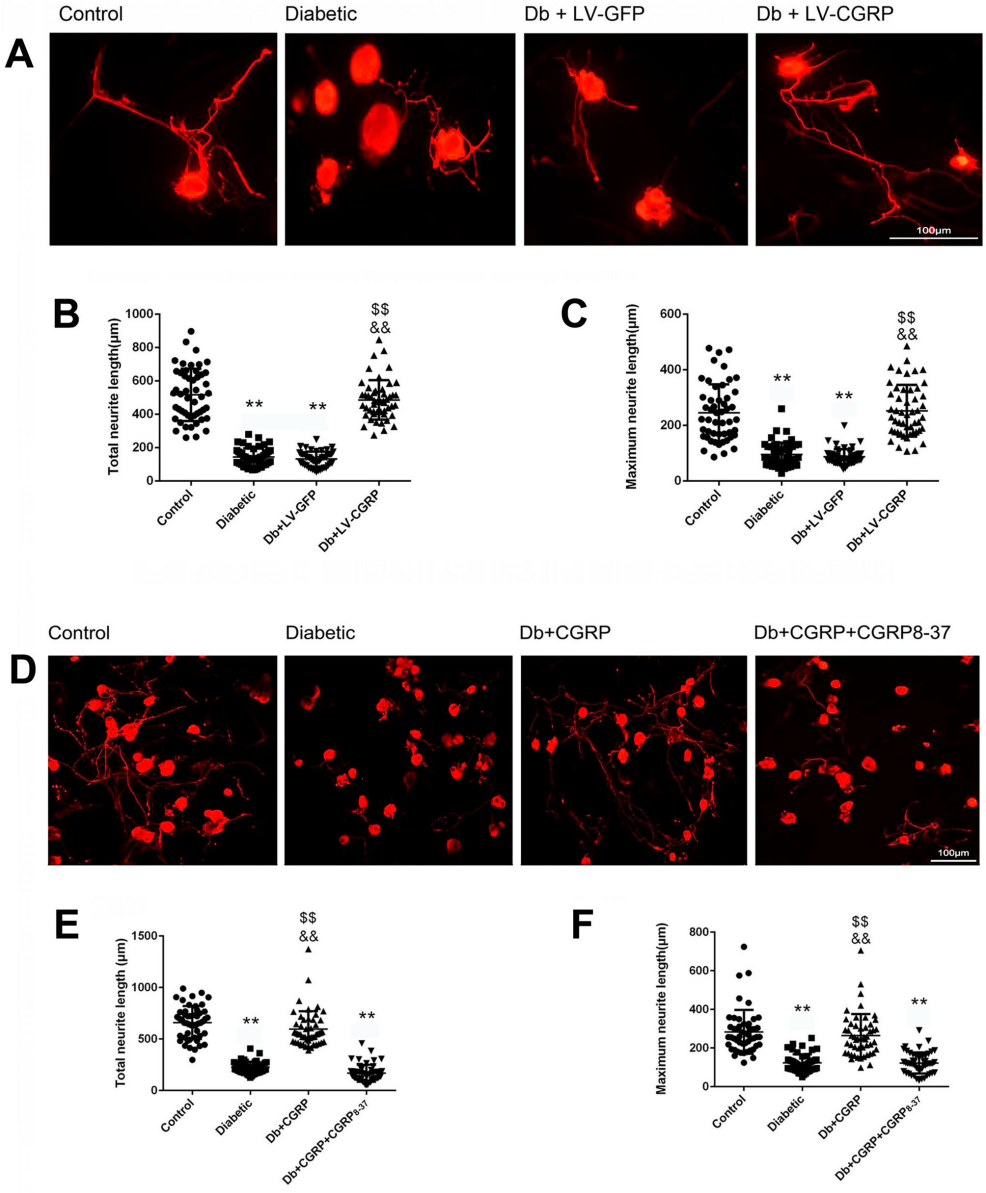
Transfection of LV-CGRP and exogenous CGRP promoted outgrowth of the neurites of the DRG neurons from the diabetic animals. (**A**) The representative images of outgrowth of the neurites of the dissociated DRG neurons from the age matched non-diabetic animals (Control), the neurons of diabetic animals without transfection of vectors (Diabetic), the neurons of diabetic animals transfected by LV-GFP (Db + LV-GFP) and by CGRP genes (Db + LV-CGRP). (**B**) Transfection of LV-CGRP fully corrected the impairment in the outgrowth of the neurites of the neurons from diabetic animals, evaluated as total neurite length (**B**) and maximum neurite length (**C**). The representative images of outgrowth of the neurites of the neurons from the non-diabetic and diabetic animals were shown in (**D**), indicate an effectiveness of CGRP (at 10^−8^ mol/L, the final concentration) on promotion of the outgrowth of the neurites of the neurons from the diabetic animals (**D**–**F**). CGRP_8–37_ completely abolished the protective effects of CGRP, suggests the specific receptor mediated the promoting actions of CGRP. **p < 0.01, compared to the control; ^$$^*p* < 0.01, compared to Diabetic group; ^&&^*p* < 0.01, compared to Db + LV-GFP group or Db + CGRP + CGRP_8–37_ group. The scales = 100 µm.

For each of the experimental groups, 50 DRG neurons collected from the 5th lumbar and sacral spinal segments of 6 ~ 7 animals were evaluated. The DRG neurons from the diabetic animals exhibited impaired neurite outgrowth, with a significantly shorter total neurites length (Control vs Diabetic, 669.7 ± 179.9 μm vs 225.5 ± 55.6 μm, *p* < 0.01) and the maximum neurite length (Control vs Diabetic, 283.8 ± 113.4 μm vs 122.8 ± 44.7 μm, *p* < 0.01, Fig. 7A–C), after 4 days of culture following the dissociation. Significant improvements were observed in the neurons transfected by LV-CGRP, in the total neurite length (Db + LV-CGRP vs Diabetic, 485.6 ± 118.8 μm vs 142.6 ± 52.6 μm, *p* < 0.01) and the maximum neurite length (Db + LV-CGRP vs Diabetic, 252.4 ± 92.8 μm vs 94.6 ± 43.0 μm, *p* < 0.01, Fig. 7A–C).

Exogenous administration of CGRP (at 10^–8^ mol/L) induced a similar protective effect showing significant promotion of the regeneration of the neurites, in the total neurites length (Db + CGRP vs Diabetic, 567.2 ± 178.4 μm vs 225.5 ± 55.6 μm, *p* < 0.01) and the maximum neurite length (Db + CGRP vs Diabetic, 265.1 ± 111.5 μm vs 122.8 ± 44.7 μm, *p* < 0.01, Fig. 7D–F). Involvement of the specific CGRP receptor mediating the protective effect was shown by complete abolition of the action by CGRP_8–37_.

### CGRP induced homeostasis of ROS, MMP in the neurons

**Figure 8:**
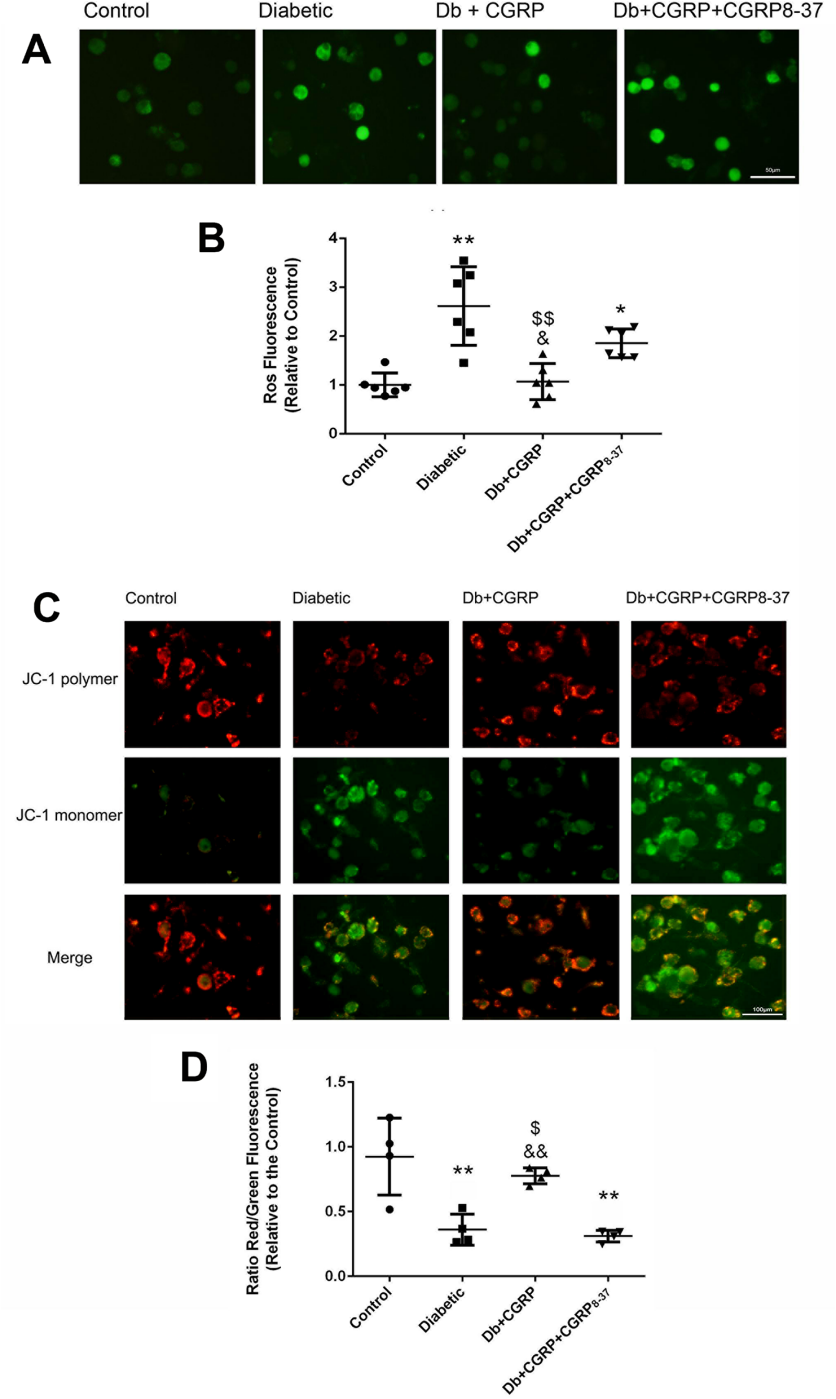
CGRP attenuated over-expression of ROS and the reduction of MMP. (**A**) The representative images of the expressions of ROS in the DRG neurons in groups of control, Diabetic, Db + CGRP and DB + CGRP + CGRP_8–37_, indicate an effective and specific receptor mediated anti-oxidative action of CGRP (**A**,**B**). The representative images in figure (**C**) show the changes in mitochondrial transmembrane potentials (MMP) in the DRG neurons, indicating the declined MMP in the neurons from the diabetic animals (Diabetic) and an effective and specific receptor mediated action of CGRP in reversion of the impairment of the MMP in the diabetes insulted neurons (**C**,**D**). *Control* neurons from the age matched non-diabetic animals, *Db + CGRP* the neurons collected from the diabetic animals, treated with CGRP, *Db + CGRP + CGRP*_*8–37*_ the neurons collected from the diabetic animals, treated by CGRP_8–37_ then by CGRP. *p < 0.05, compared to the control; **p < 0.01, compared to the control; ^$^*p* < 0.05, compared to diabetic group; ^$$^*p* < 0.01, compared to Diabetic group; ^&^*p* < 0.05, compared to Db + CGRP + CGRP_8–37_ group; ^&&^*p* < 0.01, compared to Db + CGRP + CGRP_8–37_ group. The scales = 50 µm in (**A**) and 100 µm in (**C**), respectively.

We investigated the underlying mechanism of the protective effects of CGRP in homeostasis of mitochondrial function by analysis of the alterations of intracellular ROS and mitochondrial transmembrane potential. A significantly higher level of ROS was observed in the DRG neurons from the diabetic animals (Control vs Diabetic, 1.0 ± 0.2 vs 2.5 ± 0.9, *p* < 0.01), while a significantly lower levels of ROS were seen in the neurons of the diabetic animals treated with CGRP, at a final concentration of 10^−8^ mol/L (Diabetic vs Db + CGRP, 2.5 ± 0.9 vs 1.0 ± 0.3, *p* < 0.01). The inhibitory effect of CGRP on ROS was completely abolished by CGRP_8–37_ (Fig. 8A,B).

JC-1 staining was used to detect the disruption of mitochondrial transmembrane potentials, which could be one of the early events activating apoptosis via a mitochondrial pathway. Mitochondria normally emit red fluorescence with JC-1 staining, while the decreased ratio of red to green fluorescence indicates a reduction of mitochondrial transmembrane potential. In this study, an increase in green fluorescence in DRG neurons from diabetic animals was detected, indicating depolarization of mitochondrial membrane. Exogenously administered CGRP (at 10^−8^ mol/L) restored the mitochondrial transmembrane potentials in the neurons from diabetic animals. CGRP_8–37_ (at 10^−7^ mol/L) completely abolished the effect of CGRP (Fig. 8C,D), indicating a specific receptor mediated protective action of CGRP.

## Discussion

We designed this study to investigate the correlation between desensitization of the nociceptive thermal sensation and the reduction of CGRP in peripheral sensory nerves and the mechanism underlying the pathology, by using the experimental diabetic animals. The first clue indicating a role of CGRP in the neuroprotection was the observation showing the desensitization of the noxious thermal sensation of the diabetic animals, the reduction of the number of CGRP/TRPV1 expressing small sensory neurons in the DRG and the significant reductions of CGRP, SP, NGF and TRPV1 in the ganglion neurons and in the spinal dorsal horn, which are either the nociception-related neurons or the nociception-related modulators. The changes in the molecules in the spinal cord are similar to the previous reports by Fuchs and colleagues^19^, showing significant reductions of the basal and (capsaicin) stimulated levels of CGRP and SP in peripheral nerves of diabetic rats, which may indicate impeded axonal transport. Moreover, a significant impairment in the capacity of production of CGRP, SP, NGF and TRPV1 in the residual (cultured) DRG neurons collected from the diabetic animals was detected as significantly lower levels of the proteins and the coding mRNAs of the molecules, compared with the equal amount of the DRG neurons from the non-diabetic control animals (Figs. 4C,D and 5). The greater magnitude of loss of CGRP in the spinal cord than that in the DRG may indicate a contribution of reductions of the projections of the CGRP^+^ neurons, in the spinal cord. The simultaneous the downregulations of TRPV1 and NGF, both of which may physiologically upregulate CGRP in the sensory neurons^10^, provide logical explanations why CGRP was declined in the diabetic animals. NGF plays an important role in maintenance of neural metabolism and viability of sensory nerves^20,21^. Loss of TRPV1, as a molecular nociceptor, may directly result in the desensitization of the noxious thermal sensation as observed in this study. CGRP and SP participate in modulation of nociceptive signals in the spinal cord^9,10^ while CGRP also facilitates nerve signal transmission via modulation of Schwann cells^17,18,22,23^. The reduction of TRPV1, NGF, SP and CGRP may be related to the impairment of the sensory neurons and the nociceptive sensation of the diabetic animals. Then we tried to limit the progress of the sensory desensitization by reverse of the change in CGRP and SP via chronic stimulation of TRPV1, a non-specific cation channel whose activation leads to release of CGRP and SP. Oral administration of capsaicin was conducted, based on previously findings from other and our group, showing variable doses of capsaicin, from 0.01 to 150 mg/kg/day, have been used for beneficial effects in pharmacological experiments^15,24,25^. Kawada and colleagues reported that within 60 min of administration of capsaicin into the stomach, jejunum, and ileum in the rats, approximately 50, 80, and 70% of the capsaicin had been absorbed, respectively^26^. We recently reported that TRPV1 and CGRP in the DRG were significantly normalized in the diabetic animals taking hot pepper (containing 0.017% of capsaicin) mixed in the basic diet, while the diabetic controls taking conventional laboratory food suffered significant reduction in TRPV1 and CGRP^15^. In current study, 0.017% of capsaicin mixed in the food were taken by the animals, presenting the significant normalization of the thermal sensation in both the diabetic and non-diabetic animals, while there was no desensitization of TRPV1 and the neurons expressing it. This finding is similar to the observation reported by Motte et al., showing capsaicin-enriched diet ameliorates autoimmune neuritis in experimental animal model^24^. However, opposite effect of capsaicin (at larger doses, via parenteral administration) on peripheral nociceptive neurons was found that systemic treatment with capsaicin led to degeneration of a subpopulation of small-to-medium sized primary sensory neurons and the unmyelinated axons in experimental rats by other^27–29^ and our group^13^. The findings may indicate that the biological effects differ depending on the dose, application form, application route and the bioavailability of capsaicin^30,31^, which may make the differences in the concentrations of c44apsaicin in the matrix around the neurons and the outcomes. As the possible explanations, we note that a significantly larger dose of capsaicin (33 times greater than that in current study) was injected subcutaneously, while a much smaller dose of capsaicin (3 mg/kg) was given in the food (as dietary supplement) in this study. The other difference is that the recipients were developing diabetes with degeneration of peripheral sensory nerves in this study, while the animals were healthy before given the larger dose of capsaicin in the previous report^27^. And another difference is the timing of evaluation of the nervous alteration in the previous report, at 1–6 h after the capsaicin treatment, but in current study, at a weekly interval for 8 weeks. Obviously, the differences in the dosage/route of administration of capsaicin, in the timing of evaluation of the nervous impairment and in the recipients may contribute to the different consequences of systemic capsaicin treatment. Lukacs and colleagues reported that distinctive changes in phosphoinositides underlie differential regulation of TRPV1 in nociceptive neurons^32^. Activation of phospholipase C (PLC) induces sensitization of TRPV1. Massive down-regulation of PLC in the DRG of the diabetic animals was observed by Tie-Jun Sten Shi and colleagues^33^, which may underlie the desensitization of peripheral sensory nerves in the diabetic animals. Furthermore, Lukacs and colleagues observed that maximal stimulation of TRPV1 (by larger dose of capsaicin) inhibited TRPV1, while capsaicin induced activation of TRPV1 when lack of PLC (δ4)^32^. The observations provide the explanation on the difference of desensitization of TRPV1 in previous experiments or sensitization of TRPV1 by capsaicin in this study.

It was noted, in this study, that the animals avoided the food in the first days and then became acquainted, which might indicate the concentration of capsaicin in the food was high enough to be tasted stimulating (hot and spicy) to the animals, while a rapid desensitization of the local trigeminal nerve endings in the oral mucosa might occurred under direct stimulation by the capsaicin in the food, resulting in the later increase in food consumption (Fig. 1C,D). As expected, here in this study capsaicin depolarized neurons expressing TRPV1 and hence chronic exposure to it significantly normalized the sensitivity to the noxious thermal sensation, the amount of TRPV1, CGRP, SP and NGF (as shown in Fig. 3A,C,E,G) and the small neurons expressing TRPV1 and CGRP in the animals taking capsaicin (Fig. 2F), suggesting that the treatment with capsaicin normalized the sensitivity of TRPV1 and the sensory neurons expressing it. Furthermore, we found that chronic application of capsaicin fully restored CGRP and SP in the DRG and significantly upgraded CGRP with unchanged (downregulated) SP in the spinal cord in the diabetic animals (Fig. 3B,D). The findings may suggest that chronic capsaicin stimulation is an efficient way to preserve the axonal transport for CGRP but not for SP in the sensory neurons, similar to the finding reported by Robinson and colleagues^34^, which suggested a focus on CGRP in the next step of this study.

The novel finding of this study reveals an independent neuroprotective role of CGRP in the DPN, by demonstration of the restoration of CGRP completely reversed the impaired and reduced survival of the neurons and ability of outgrowth of the neurites without upregulation of SP and NGF in the neurons, after transfection of LV-CGRP or after exogenously administered CGRP. The findings indicate that preservation of the CGRP and the small DRG neurons may also contribute to the neuroprotection. As disturbance in metabolism of ROS in the mitochondria has been indicated important in the pathology of diabetes^5,7,16–18^, alterations of ROS and mitochondrial membrane potential were evaluated in this study. The normalization of the metabolism of ROS and the mitochondrial transmembrane potential by the same experimental settings achieved in this study may indicate the mechanism underlying the neuroprotective effects of CGRP and the normalization of the metabolism of ROS and the mitochondrial transmembrane potential. The finding, in this study, that the protective effects of exogenously added CGRP were blocked by CGRP_8–37_, an antagonist of the standard membrane-bound CGRP receptor, not only confirms the specific receptor mediated the effects of CGRP, but also indicates how capsaicin produced the protective effects in the animals, ie, the CGRP exerts the neuroprotection, after released from the neurons following activation of TRPV1. The protective effects of the endogenous CGRP or the exogenously supplemented CGRP are mediated by the specific receptors on the neuron somata and its central terminals but would require an unknown Schwann cell mediation at peripheral terminals to become effective^35^.

The surprising novel finding of this study is the synchronous normalization (upregulation) of TRPV1 in the DRG of the diabetic animals taking capsaicin (Fig. 3G) and in the cultured DRG neurons from the diabetic animals, but transfected with LV-CGRP (Fig. 5A–C). The findings expand the finding of the role of αCGRP in TRPV1 mobilization to the neuronal surface in peptidergic nociceptors^36^. The important finding may indicate a positive feedback action of CGRP to secure the homeostasis of the TRPV1 in the DRG neurons, by which maintenance of the normal regulation of the level of CGRP is enforced in the neurons. The finding indicates that modulation of endogenous CGRP may be an effective way to rebuild the loop of cross-enforcing mechanism between TRPV1 and CGRP in diabetic neuropathy and the enteral administration of capsaicin may be a safe and convenient way for the chronic intervention. The observations also explained how chronic treatment with capsaicin upregulated TRPV1 in the dorsal root ganglion neurons of the diabetic animals, observed in this study (Fig. 3G).

The finding suggests that an independent neuroprotective effect of CGRP, because there was no any agonist of TRPV1 added in the culture of the neurons in the study, although TRPV1 was simultaneously upregulated up to the normal level by the CGRP-gene transfection. This finding may suggest CGRP not only plays a role as an important transmitter of nociceptive signals but also plays an important role in protection and neurotrophy in the peripheral nervous system. However, the observations of improvement of the viability of the cultured DRG neurons and the outgrowth of neurites may indicate that preservation of the CGRP and the small DRG neurons expressing it may also contribute to the neuroprotection, although the equal proportion of the small neurons immunoreactive for and not for TRPV1 and CGRP in the DRG.

This study revealed that capsaicin depolarizes neurons expressing TRPV1 and hence chronic exposure to this neurotoxin induces increase in extracellular CGRP and a tonic increase in CGRP receptor activity, which may act to alter CGRP and TRPV1 expression in both neurons that normally express them and in neurons that do not. Activation of the CGRP receptor modifying ROS and mitochondrial membrane potential is entirely consistent with this scenario. The findings of current study may demonstrate the neuroprotective effects of CGRP in the normalization of the proportion of TRPV1/CGRP expressing neurons and also the probability of correction of the impaired viability of the DRG neurons, the outgrowth of the neurites and the axonal transport function in the development of diabetes via normalization of the metabolism of ROS and the mitochondrial transmembrane potential.

There are limitations in current study. Lacking suitable control groups in the in vitro experiment, i.e. the control + LV-CGRP, and functioning changes in mechanical perception or nerve conduction or structural abnormalities of peripheral nerve weakened the convincement of the findings. The total number of non-peptidergic neurons in each DRG was not counted, in which the non-peptidergic small DRG neurons may also be nociceptive and need to be investigated in future study, although the work reported here focused on the peptidergic small DRG neurons. The cell injury was evaluated using TUNEL assay only but should have been confirmed by staining with cleaved caspase 3 and the changes in amount of transporting CGRP and TRPV1 in the neurites^36,37^ were not analyzed. All of these may limit comprehensive judgement of the interventional effects.

## Conclusions

Little is known about the role of calcitonin gene-related peptide in the maintenance of viability and homeostasis of peripheral sensory neurons physiologically and in pathological conditions. Here the findings in this study indicate an efficient and beneficial effect of capsaicin by enhancing the CGRP content in the sensory neurons that plays a critical role in attenuation of the mitochondrial dysfunction by correction of the abnormality of ROS in the DRG neurons of the diabetic animals, which makes predominant contributions in the preservation of the viability of the neurons and the outgrowth of the neurites. Diminishing the expression of CGRP may play a critical role in the pathogenesis of the diabetic peripheral neuropathy, avoidance or correction of which may be an applicable and effective way to prevent or to intervene in the sensory nerve impairments.

## Methods

### Animals

Healthy male Sprague–Dawley rats, 8–9 weeks of age (weighting 250–270 g, provided by the Experimental Animal Laboratory of Shanxi Medical University) were employed in the in vivo and the in vitro experiments of this study. The animals were housed at a maximum of four per cage under a 12-h light/12-h dark cycle with access to food and water ad libitum. The housing of the animals and the experiment procedures were carried out in accordance with the Guide for the Care and Use of Laboratory Animals (United States National Institutes of Health) and were approved by the Ethical Committee for Care and Use of Laboratory Animals of Shanxi Medical University.

### Preparation of the diabetic model

The animals assigned to diabetic group were treated with intraperitoneal injection of STZ at 50 mg/kg (dissolved in 0.1 mol/L citrate buffer at pH 4.5) after 16 h fasting and evaluated as we previously reported^14,15^. The blood glucose was measured using a OneTouch Ultra blood glucose meter (Optium, Abbott, USA). When a reading of ‘Hi’ was given, the blood sample was diluted for 10 times with 0.9% saline and then measurement was carried out again. The reading was recorded as the concentration of blood glucose in this study. Diabetes was defined by a sustained blood glucose concentration greater than 16.7 mmol/L 24 h after the injection of STZ^38^, while those showed the blood glucose less than 16.7 mmol/L were excluded from the further experiment. After the treatment, the blood-glucose, the body weight and the tail flick latency (TFL), a threshold of response to noxious heating stimulation, were measured at a weekly interval for 8 weeks. The diabetic animals were randomly assigned to two subgroups, one was supplied with the food containing 0.0174% (w/w) of capsaicin (Xi’An Bosheng biological technology Co. Ltd., China), as Db + Cap group, while another group was given conventional laboratory chew, as Db group. Two groups of age-matched non-diabetic animals were enrolled as a non-diabetic control (Control group) and a non-diabetic capsaicin-treated control group (Ctrl + Cap group). Dietary capsaicin started one week before the STZ treatment (Fig. 1A). At the end of 8 weeks after the treatment with STZ, tissue samples were collected for the analyses following euthanasia of the animals, by intravenous injection of sodium pentobarbital (260 mg/kg, Sigma).

### Measurement of tail flick latency in the animals

The sensory nerve impairment was functionally evaluated by measurement of tail flick latency in the animals, using the radiant heater (37,360, UGO BSILE, Italy), setting at 40 mW/cm^2^, which induces a baseline TFL value of 14.4 ± 1.0 s, before injection of STZ. A cut-off time of 30 s was set to prevent tissue damage, as previously reported^14^.

### Examination of DRG neurons

We dissected the DRG neurons and the spinal cord from the animals 8 weeks after the treatment with STZ and then performed two parts of the experiments. First, we evaluated the changes in the number of the TRPV1 and CGRP co-expressing neurons and the expressions of TRPV1 and CGRP in the spinal dorsal horn. Then we quantitatively analyzed the amount of CGRP, SP, NGF and TRPV1 in the DRGs and the spinal cord in the diabetic animals. Second, we dissociated and cultured the DRG neurons from diabetic and non-diabetic animals and evaluated the viability of the soma, the regeneration of the neurites of the neurons and amount of LDH in the culture medium of the dissociated cultured DRG neurons as the readout for cell injury. The intracellular reactive oxygen species and mitochondrial transmembrane potentials were also evaluated.

#### Immunohistochemistry assay and imaging

The Immunohistochemistry assay and imaging were carried out according to previous reports^15,39^. We transcardially perfused the animals, using 4% paraformaldehyde in 0.1 M phosphate buffer, under deep anesthesia (with sodium pentobarbital, Sigma, 130 mg/kg, via intraperitoneal injection). Spinal cord and bilateral dorsal root ganglia (DRG) at the 5th lumbar and the first sacral segments were dissected and fixed in 4% of phosphate-buffered paraformaldehyde (for 16 h) then incubated in 30% sucrose overnight. The specimen was embedded in Optimal Cutting Temperature medium (OCT, SAKURA Tissue-Tek, Torrance, CA, USA) and sectioned at 10 μm using a cryostat (Leica CM 1850, Nussloch, Germany). The sections were washed with 0.1 M PBS (pH 7.4) and incubated with 3% H_2_O_2_ (for 20 min), then the sections were blocked with 10% normal goat serum, 0.4% bovine serum albumin and 0.3% Triton X-100 in PBS (for 1 h at room temperature). The sections were incubated with mouse anti-CGRP primary antibody (1:500, Abcam, Cambridge, UK) and rabbit anti-TRPV1 (1:500, Abcam, Cambridge, UK) and then incubated with Alexa Fluor 568 (conjugated with goat anti-mouse, presenting CGRP in red color) and Alexa Fluor 488 (conjugated with donkey anti-rabbit, presenting TRPV1 in green color, Invitrogen, Oregon, USA), at a dilution of 1:1000. The sections were examined and photographed using a microscope (Olympus, BX51), equipped with epifluorescence illumination. The negative control was carried out by omitting the primary anti-body with other procedures the same as described above, to evaluate the specificity of the stains. To minimize artificial or subjective impact on the analysis, attention was paid to limit the variations of intra- and inter-testing in processing the staining conditions of the sections from different groups. A positive criterion was clearly established in each of experiments via processing a set of sections of different treatments (groups).

#### Stereological counting

According to previous report^39^, we used a computer-aided multi-mode automatic scan system, Cytation 5 (BioTek, USA) to aid the cell counting in this study. Six DRGs were collected from the 5th lumbar and the first sacral segment, from 6 animals for each experimental group. Two of the maximal Sections (10 µm in thickness) of each ganglion were scanned. The results obtained from each DRG, the two readings, were averaged (into one point). Contours of the region were drawn at 10 × magnification (air immersion). Quantifications were performed at 40× (air immersion) using a counting frame of 70 μm × 70 μm, grid size set to 150 × 150 μm, with a guard zone of 2 μm and dissector height set at 10 μm. The sections of the DRG were scanned and the numerical density of the fluorescence was digitized simultaneously and automatically. The neurons co-expressing TRPV1 and CGRP were manually counted after merging the scanned images.

### Neuronal injury

The neuronal injury of peripheral sensory neurons in diabetes was evaluated in dissociated, cultured dorsal root ganglion neurons collected from the diabetic animals at the end of the 8 weeks after injection of STZ, by examine the abnormalities in the viability of the soma and the regeneration of the outgrowth of the neurites of the neurons.

#### Dissociation and culture of DRG neurons

The dorsal root ganglia were dissected from the cervical, thoracic, lumbar and the sacral spinal segments of the diabetic animal at the end of the 8 weeks after the injection of STZ and from the age-matched non-diabetic animals, after euthanasia. The ganglia were removed aseptically from each animal and collected in Hank’s balanced salt solution (HBSS). After being dissected into small pieces, the ganglia were digested in collagenase type II (1.5 mg/ml, Gibco) at 37 °C for 2 h, followed by 0.05% trypsin–EDTA (Invitrogen) for 30 min at 37 °C. The tissues were triturated with a fire-polished thin pipette in Dulbecco’s Modified Eagle Medium/Nutrient Mixture F-12 media (DMEM/F12, Hyclone), containing 10% fetal bovine serum, FBS (Invitrogen), penicillin (100 U/ml) and streptomycin (50 μg/ml, Invitrogen). The cells were subjected to density gradient centrifugation at 400 Ug for 10 min and then were resuspended in DMEM. The cell suspension was filtered through a cell strainer (75 µM, Becton Dickinson Labware, NJ, USA). Then the cells were seeded in poly-d-lysine coated plates (6 and 96 well plastic tissue culture plates) at a cell density of 3 ~ 5 × 10^4^/ml cells per well, in presence of 35 mM of glucose for the neurons collected from the diabetic animals and 17 mM of glucose for the neurons from the non-diabetic animals, in a humid incubator at 37 °C with 5% CO_2_ and 95% air, according to modified previously reported method^40^.

#### TUNEL assay

The cultured cells on the coverslips, from each of the 6–8 animals per group were examined. DNA fragmentation in the neurons was detected by using a TUNEL assay kit (Roche, Germany), as we previously reported^13,14,41^. Five microscopic images (× 40) were randomly photographed for each coverslip and analyzed using Image J. The number of TUNEL-positive cells was counted. Apoptosis index was the ratio of TUNEL-positive cells to the total cells observed.

#### Neurite growth assessment

We analyzed the outgrowth of the neurites in dissociated and cultured neurons from the diabetic and non-diabetic animals to evaluate the impairment of the sensory nerves and the improvement of the neuronal injury. The cultured DRG neurons on the coverslips from 6–8 animals per group were fixed for 30 min in 4% paraformaldehyde (0.1 M phosphate buffer, pH 7.4) and processed for dying with the primary rabbit anti-beta III tubulin (1:500, Abcam, USA) and the secondary antibody anti-rabbit IgG Alexa Fluor-594 (1:1000; Invitrogen, Italy). The imaging was carried out with an Olympus fluorescence microscope (BX51, Olympus, Japan). The quantified parameters, the total neurite length (sum of length of all neurites) and maximum neurite length (length of the longest neurite) were analyzed using the Image J software with the Neuron J Plugin^42^, via identifying cell bodies and tracing all neurites associated with each cell body. For each experiment of the six individual tests for each group, 50 individual neurons from 6–8 animals were evaluated.

#### Lactic dehydrogenase (LDH)

The cultured cells from each of the 6–8 animals per group were involved in the tested. LDH in the culture medium was measured using a Cytotoxicity Detection Kit (Dojindo, Japan), as we previously reported^14,43^. The amount of LDH was assessed by measuring optical absorbance using a microplate reader (Thermo Fisher) at 490 nm and presented as fold of control.

### Intervention of neuronal injury

We tested to intervene the neuronal injury in the dissociated and cultured dorsal root ganglion neurons. We restored the CGRP by up-regulating CGRP genes using an over-expressing vector containing CGRP coding genes. And we also tested efficacy of a potential pharmacological intervention by using exogenous administration of CGRP.

#### Lentivirus (LV)-mediated CGRP gene transfection

The lentiviral vectors/GV358 encoding CGRP (NM_001033953) and green fluorescent protein (GFP) were purchased from a commercial supplier (SHANGHAI GENECHEM CO., LTD., Shanghai, China). The sequence of genetic tags in the vector was Ubi-MCS-3FLAG-SV40-EGFP-IRES-puromycin. On the second day of culturing, the DRG neurons from the diabetic rats were transfected with the lentivirus-CGRP or the lentivirus-GFP (as a control) at a multiple of infection (MOI) of 100 for 16 h and then the culture medium was replaced by the conventional medium. Then the neurons were cultured for two more days (as presented in Fig. 4A). The initial success in the gene transfection was confirmed by visualization of the green proteins in the neurons. Real-time quantitative PCR (qRT-PCR) and ELISA were used for the quantitative analyses of the alterations of CGRP coding mRNA and CGRP, to evaluate the efficiency of the gene transfection.

#### Pharmacological intervention

To test the feasibility of a pharmacological option to improve the impairment of the ganglion neurons dissociated from the diabetic animals, CGRP (at a final concentration of 10^–8^ mol/L) alone and with its specific antagonist, CGRP_8–37_ (at a final concentration of 10^–7^ mol/L, given at 30 min before use of CGRP) were added in the culture medium, co-culturing the neurons for 48 h, according to our previous studies^14,16^ but with modifications in the timing of delivery of CGRP_8–37_ and the incubation time.

### Measurement of changes of molecules

#### Quantification of TRPV1

We quantitatively analyzed TRPV1 using Western blotting assay in this study. The DRGs from the lumbar and the sacral spinal segments of 6 animals per group (n = 6) were collected and pulverized in liquid nitrogen and homogenized at 4 °C with cold lysis buffer (150 mM NaCl, 20 mM Tris–HCl, pH 7.4, 1% NP-40, 10 μg/ml leupeptin, 20 μg/ml aprotinin), and then centrifuged at 15,000*g* for 20 min at 4 °C. The supernatants were collected and immediately frozen at − 80 °C for later analysis. While six wells of the cultured neurons (from 6 ~ 8 animals) per group were homogenized in RIPA lysis buffer with protease inhibitor (Boster, China). The samples were centrifuged at 10000*g* for 15 min and the supernatant was collected for the assay.

The total protein in the samples were measured using the BCA Protein Assay kit (Thermo, Rockford, IL, USA). After boiling at 95 °C for 5 min, 50 μg of whole protein extract from each sample were loaded into 10% SDS-PAGE gels for electrophoresis (60 V/30 min followed by 150 V/60 min) and then transferred to PVDF membrane, at 60 V for 70 min using a wet-transfer system (MINI TRANS-BLOT CELL, Bio-Rad Laboratories, Inc., CA, USA). Thereafter, the membrane was blocked with 3% skim milk dissolved in PBS for 2 h at room temperature, and then incubated with primary antibodies of TRPV1 (1:2000, Abcam, Cambridge, MA, USA) and GAPDH (1:2000, Beijing Biosynthesis Biotechnology Co., LTD. Beijing, China) overnight at 4 °C. The membrane was washed with PBST (0.05% Tween-20 in PBS) and then incubated with a secondary HRP conjugated antibody (1:1500, Santa Cruz Biotechnology, Inc., CA, USA) at room temperature for 2 h. The ECL chemical luminescence method was employed to detect the labeled antigens. The target proteins were detected and analyzed using the Quantity One imaging software (Bio-Rad Laboratories, Inc., CA, USA) and standardized in comparison with GAPDH, as we previously reported^15^.

#### Quantification of CGRP, SP and NGF

Quantitative analysis of the amount of CGRP, SP and NGF were carried out using the enzyme-linked immunosorbent assay (ELISA). The spinal cord and DRGs from the 5th lumbar and the sacral spinal segments from each of the six animals per group (n = 6) were collected and pulverized in liquid nitrogen and homogenized and then centrifuged at 15,000*g* for 20 min at 4 °C. The supernatants were collected and immediately frozen at − 80 °C for later analysis. The alterations of the CGRP, SP and NGF in the cultured DRG cells were also analyzed using ELISA method. Six wells of the cultured cells (from 6–8 animals) per group were homogenized in RIPA lysis buffer with protease inhibitor (Boster, China). The samples were centrifuged at 10000*g* for 15 min and the supernatant was collected for the assay. The concentration of the protein in the samples were measured using the BCA Protein Assay kit (Thermo, Rockford, IL, USA). Analysis was performed using the Enzyme-linked immunosorbent Assay kits (Tianjin Anoric Biotechnology, China), with the ranges of detection shown: CGRP, 0–20,000 pg/mL, SP, 0–2500 pg/mL and NGF, 0–10,000 pg/mL. Calibration solutions and buffers were prepared according to the instructions. Supernatants were thawed and samples were prepared to a concentration of 100 μg of protein in a volume of 50 μL. Diluent from the kit was added to make up the sample to 100 μL, then samples were loaded onto the reading plate, incubated, and washed according to the manufacturer’s protocol. The plate was then read on the Multiskan Ascent (Thermo, USA), as we previously reported^13,15,44^. The readings obtained were normalized to total protein concentrations and presented as pg/mg protein.

#### Quantification of coding mRNA

The coding mRNA of TRPV1, CGRP, SP and NGF in the dissociated and cultured dorsal root ganglion neurons were evaluated using real-time-RT-PCR, as we previously reported^45^. Six wells of the cultured cells (collected from 6–8 animals) per group were collected and frozen in liquid nitrogen and stored at − 80 °C. Total RNA was extracted using TRIzol reagent (Invitrogen, USA) according to manufacturer’s instructions. An Agilent 2100 Bioanalyzer (Agilent Technologies, Palo Alto, CA, USA) was used to evaluate the RNA concentration and integrity to ensure the quality of the isolated RNA was adequate for further analysis. The RNA samples were used as templates for synthesis of the first strand the cDNA. Of the total RNA samples, 1 μg was used for the reverse transcription with a cDNA Reverse Transcription Kit (TaKaRa Biotechnology, Dalian, China) according to the manufacturer’s instructions. Primers were designed using the Genomatix program based on published sequences for different species in the GenBank. The primer sequences were as follows:

TRPV1 forward: 5′-GAAGCAGTTTGTCAATGCCAGCTA-3′.

TRPV1 reverse: 5′- AGGGTCACCAGCGTCATGTTC-3′.

CGRP forward: 5′-ATGGGCTTTCTGAAGTTCTCC-3′.

CGRP reverse: 5′-GGCCTGCTTTCCAAGGTTGAC-3′.

SP forward: 5′-GAGCCCTTTGAGCATCTTCT-3′.

SP reverse: 5′-ACGCCTTCTTTCGTAAGTTCTG-3′.

NGF forward: 5′-CTCATCCACCCACCCAGTCTT-3′.

NGF reverse: 5′-AGTCAGCCTCTTCTTGTAGCCTT-3′.

GAPDH forward: 5′-ACCACAGTCCATGCCATCAC-3′.

GAPDH reverse: 5′-TCCACCACCCTGTTGCTGTA-3′.

The Real-time PCR reactions were performed, using SYBR Green master mix (TaKaRa Biotechnology, Dalian, China) on a fluorescent temperature cycler (MX3005P Real-time PCR System, Stratagene, USA). GAPDH was amplified in each reaction, serving as an internal reference during quantitation, to correct the variation from individual of the animals and/or experimental variations. The mean threshold cycle (CT) values for both the targets (TRPV1, CGRP, SP and NGF) and internal control (GAPDH) genes were determined in each sample. Gene expression was normalized to GAPDH, and comparative 2−ΔΔCt method was performed for relative expression analysis.

#### Intracellular ROS

Intracellular reactive oxygen species were measured, based on the reaction of oxidation of 2′,7′-dichlorofluoresce diacetate (DCFH-DA) to a fluorescent 2′,7′-dichlorofluorescein (DCF) by reactive oxygen species in the substrates. The cultured neurons on the coverslips, from 6–8 animals per group were treated with DCFH-DA (10 μmol/l, Sigma-Aldrich, USA) for 30 min at 37 °C in the dark, as reported^17^. Then the cells were visualized and photographed using a microscope (BX51, Olympus) equipped with epifluorescence optics and a 100-W mercury lamp (Olympus BH2-RFLT3). The images were analyzed by ImageJ, a NIH software. The amount of reactive oxygen species was expressed as fluorescence intensity (FI). For each group in each of 6 individual tests, 50 individual neurons from 6–8 animals were analyzed.

#### Mitochondrial transmembrane potentials

Mitochondrial transmembrane potential was assessed using 5,5′,6,6′-tetrachloro-1,1′3,3′-tetraethylbenzamidazol carbocyanine (JC-1, Beyotime Institute of Biotechnology, China). The amount of JC-1 taken in by the mitochondria is directly related to the magnitude of the mitochondrial transmembrane potential. The greater the mitochondrial uptake, the more JC-1 in aggregate forms (in red fluorescence) presents, oppositely to the JC-1 monomer that fluoresces in green color^16,46^. The cultured cells on each of the coverslips from 6–8 animals per group were tested. The staining solution was freshly prepared by diluting the stock solution (at 1:400) in warm culture medium (at 37 °C) supplemented with 10% calf serum. Two milliliters of the staining solution were immediately applied to the neurons grown on a coverslip in each well, after drainage of the culture medium. Then the neurons were incubated for 20 min, and then were immediately analyzed after rinse with warm dye-free culture medium. For evaluating the fluorescence of JC-1, a manual model was used with the same setting for the microphotography of the green and red fluorescence of JC-1, the monomers and aggregates respectively, during intra-assay. To compare the transmembrane potentials of the mitochondria between the groups, the intensity of JC-1 fluorescence was measured using ImageJ software. Average pixel intensities inside the contours of individual mitochondrion were determined. For analysis of the alterations in mitochondrial transmembrane potential, data from 75 cells were collected under high power view of microscope (100 × magnification, oil immersion) in each of 6 experiments and the mean values of fluorescence intensity (FI) and the deviations were calculated, as previously reported^47^. The intra- and inter-assay variations in the FI among the coverslips in the same group underwent the same treatments were evaluated for the quality control of the experiments. A higher red/green ratio indicates a more polarized, or more negative and hyperpolarized mitochondrial inner membrane. For each group in each experiment of 6 individual tests, 75 individual neurons from 6–8 animals were involved.

### Statistical analysis

Tail flick latency, the neuron density in the DRG, the viability and outgrowth of neurites were analyzed using two-factor ANOVA, with diabetes and treatment as factors. For biochemical analysis, another cohort of the animals in the controls, the diabetes and the treatment(s) groups, at least six animals in each group, in the experiments including that using dissociated and cultured neurons collected from the control and diabetic animals were used. Analysis via a linear mixed effects model with a random intercept for experiment revealed that the results and effect estimates were negligibly sensitive to experimental differences. Peptide profiling of CGRP, SP and NGF in the DRG and the cultured neurons, and LDH in the culture medium were analyzed using a gamma mixed-effects model with random intercepts for each animal due to the distribution of the data. The data were analyzed with GraphPad Prism 6 (San Diego, California, USA). All values were expressed as mean ± standard deviation (S.D.) with ‘n’ indicates the number of independent experiments performed at different preparations from at least six animals in each group in the experiments. For the data that did not pass the normality test, the nonparametric Mann–Whitney test was used to assess statistical significance. The other parameters were compared using Student's t-test for two groups and one-way ANOVA followed by post hoc Bonferroni's test for multiple groups. The statistically significance was considered when *p* value was less than 0.05.

### Ethics approval

The housing of the animals and all procedures were in accordance with the Guide for the Care and Use of Laboratory Animals (U.S. National Institutes of Health) and were approved by the Ethical Committee for Care and Use of Laboratory Animals of Shanxi Medical University. This article does not contain any studies with human participants performed by any of the authors.

## Supplementary Information

Supplementary Information 1.

Supplementary Information 2.

## Data Availability

The raw datasets generated and analyzed for this study can be viewed on request from the authors.

## Abbreviations

CGRP: Calcitonin gene-related peptide
DPN: Diabetic peripheral neuropathy
DRG: Dorsal root ganglion
ELISA: Enzyme linked immunosorbent assay
LDH: Lactic dehydrogenase
MMP: Mitochondrial transmembrane potential
NGF: Nerve growth factor
PLC: Phospholipase C
ROS: Reactive oxygen species
SP: Substance P
STZ: Streptozotocin
TFL: Tail flick latency
TRPV1: Transient receptor potential vanilloid 1
TUNEL: Terminal deoxynucleotidyl transferase (TdT) dUTP nick end labeling
