# Supplementary material for: Dietary capsaicin normalizes CGRP peptidergic DRG neurons in experimental diabetic peripheral neuropathy
Source: Sci Rep. 2021 Jan 18;11:1704. doi: 10.1038/s41598-021-81427-w (PMC7814129; doi:10.1038/s41598-021-81427-w)
Supplement: Supplementary file 1 — Supplementary Information 1. [file 41598_2021_81427_MOESM1_ESM.pdf]

**Dietary capsaicin rescues CGRP peptidergic DRG Neurons in experimental diabetic peripheral neuropathy**

Authors: Xiao-Yi Zhang[1], Zheng Guo[1,2,3,]* Tu-Ping Li[2], Tao Sun[2]

1. Department of Anesthesiology, Shanxi Medical University, 86 Xinjiannan Road, Taiyuan 030001, Shanxi, China
2. Department of Anesthesiology, Second Hospital of Shanxi Medical University, 382 Wuyi Road, Taiyuan 030001, Shanxi, China
3. Key Laboratory of Cellular Physiology (Shanxi Medical University), National Education Commission, Shanxi Medical University, 86 Xinjiannan Road, Taiyuan 030001, Shanxi, China

Emails of the authors:

Xiao-Yi Zhang, zhangxiaoyi229@163.com

Zheng Guo, guozheng713@yahoo.com

Tu-Ping Li, lititu001@163.com

Tao Sun, 572462311@qq.com

*Corresponding author: Prof. Zheng Guo, Email: guozheng713@yahoo.com

**Western blot in the in vitro experiment 1**

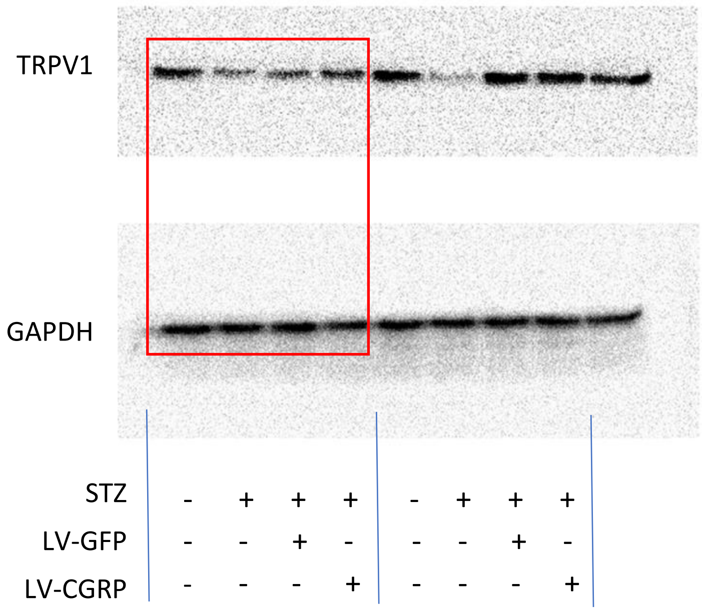


-, not added; +, added; The blots in the red box are used in Fig. 5B.

**Western blot in the in vitro experiment 2**

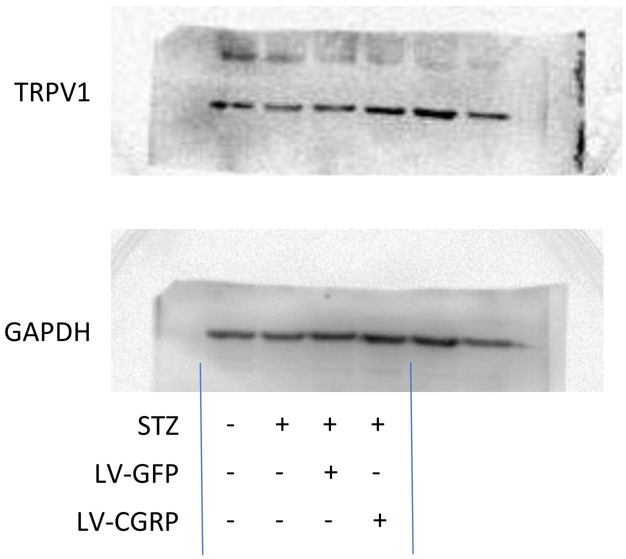


-, not added; +, added

**Western blot in the in vitro experiment 3**

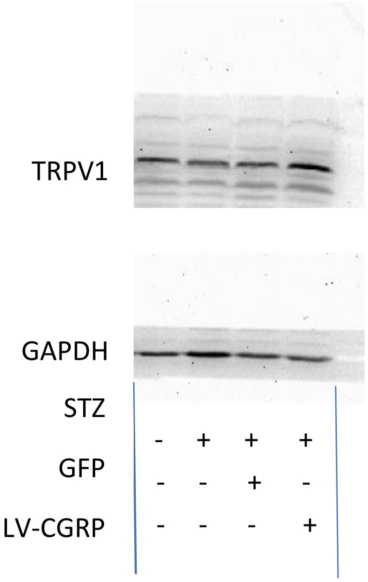


-, not added; +, added
