# Supplementary material for: Dietary capsaicin normalizes CGRP peptidergic DRG neurons in experimental diabetic peripheral neuropathy
Source: Sci Rep. 2021 Jan 18;11:1704. doi: 10.1038/s41598-021-81427-w (PMC7814129; doi:10.1038/s41598-021-81427-w)
Supplement: Supplementary file 2 — Supplementary Information 2. [file 41598_2021_81427_MOESM2_ESM.pdf]

Dietary capsaicin preserves CGRP peptidergic DRG neurons in experimental diabetic peripheral neuropathy

Authors: Xiao-Yi Zhang[1], Zheng Guo[1,2,3,] * Tu-Ping Li[2], Tao Sun[2]

1. Department of Anesthesiology, Shanxi Medical University, 86 Xinjiannan Road, Taiyuan 030001, Shanxi, China
2. Department of Anesthesiology, Second Hospital of Shanxi Medical University, 382 Wuyi Road, Taiyuan 030001, Shanxi, China
3. Key Laboratory of Cellular Physiology (Shanxi Medical University), National Education Commission, Shanxi Medical University, 86 Xinjiannan Road, Taiyuan 030001, Shanxi, China

Emails of the authors:

Xiao-Yi Zhang, zhangxiaoyi229@163.com

Zheng Guo, guozheng713@yahoo.com

Tu-Ping Li, litutu001@163.com

Tao Sun, 572462311@qq.com

***Corresponding author:** Prof. Zheng Guo, Email: guozheng713@yahoo.com

Table S1 Fluorescence intensity of TRPV1 and CGRP in DRG and spinal cord [n = 6; Mean (SD)]

	TRPV1				CGRP			
	Ctrl	Ctrl+Cap	Db	Db+Cap	Ctrl	Ctrl+Cap	Db	Db+Cap
DRG	4180.65 (155.13)	5075.67* (176.02)	2609.31* (133.87)	3944.33*& (59.91)	3603.38 (95.69)	4763.67* (175.57)	2190.00* (205.51)	3258.00*& (137.70)
SC	5735.30 (102.84)	7198.00* (171.19)	3598.36* (264.48)	5032.00*& (163.45)	5051.33 (149.23)	6855.00* (127.49)	2938.38* (56.24)	4708.65*& (138.59)

Ctrl = control; Ctrl+Cap = non-diabetic animals treated with capsaicin; Db = diabetic; Db+Cap = diabetic animals treated with capsaicin; DRG = dorsal root ganglia; SC = spinal cord; * = $p < 0.05$, compared with control; & = $p < 0.05$, compared with diabetic

Table S2 Proportion of small neurons of $TRPV1^+$/$TRPV1^-$ in the DRG [n = 6; Mean (SD)]

**$TRPV1^+$/TOSN**				**$TRPV1^-$/TOSN**			
Ctrl	Ctrl+Cap	Db	Db+Cap	Ctrl	Ctrl+Cap	Db	Db+Cap
15.6(2.6)	19.2(2.6)*	11.0(2.1)*	15.7(1.6)&#	84.4(2.6)	80.8(2.6)*	89.0(2.1)*	84.3(1.6)&#

$TRPV1^+$ = small neurons immunoreactive for TRPV1; $TRPV1^-$ = small neurons not immunoreactive for TRPV1; TOSM = total small neurons observed; Ctrl = control; Ctrl+Cap = non-diabetic animals treated with capsaicin; Db = diabetic animals; Db+Cap = diabetic animals treated with capsaicin; DRG = dorsal root ganglia; * = $p < 0.05$, compared with control; & = $p < 0.05$, compared with diabetic; # = $p < 0.05$, compared with Ctrl+Cap

Table S3 Proportion of small neurons of $CGRP^{+}/CGRP^{-}$ in the DRG [n = 6; Mean (SD)]

**$CGRP^{+}$/TON**				**$CGRP^{-}$/TON**			
Ctrl	Ctrl+Cap	Db	Db+Cap	Ctrl	Ctrl+Cap	Db	Db+Cap
21(4.2)	22.7(2.6)	16.2(0.6)*	18.6(2.4)&#	79.0(4.2)	77.3(2.6)	83.8(0.6)*	81.4(2.4)&#

$CGRP^{+}$ = small neurons immunoreactive for CGRP; $CGRP^{-}$ = small neurons not immunoreactive for CGRP; TOSM = total small neurons observed; Ctrl = control; Ctrl+Cap = non-diabetic animals treated with capsaicin; Db = diabetic animals; Db+Cap = diabetic animals treated with capsaicin; DRG = dorsal root ganglia; * = $p < 0.05$, compared with control; & = $p < 0.05$, compared with diabetic; # = $p < 0.05$, compared with Ctrl+Cap
